# Differential Regional Brain Spontaneous Activity in Subgroups of Mild Cognitive Impairment

**DOI:** 10.3389/fnhum.2020.00002

**Published:** 2020-01-30

**Authors:** Qi-Hui Zhou, Kun Wang, Xiao-Ming Zhang, Li Wang, Jiang-Hong Liu

**Affiliations:** ^1^Department of Neurology, Xuanwu Hospital, Capital Medical University, Beijing, China; ^2^Department of Neurology, Beijing Puren Hospital, Beijing, China; ^3^Department of Psychiatry, Beijing Huilongguan Hospital, Beijing, China

**Keywords:** mild cognitive impairment, resting state, fMRI, the amplitude of low-frequency fluctuation, Alzheimer’s disease

## Abstract

**Background**: Amnestic mild cognitive impairment (aMCI) has a high conversion risk to Alzheimer’s disease (AD). The aMCI patients may have only a memory deficit (single-domain-aMCI, sd-aMCI) or deficits in multiple cognitive domains (multiple-domain-aMCI, md-aMCI). However, differences in intrinsic brain activity between these two sub-types remain unclear.

**Method**: Neuropsychological and resting-state functional magnetic resonance imaging (fMRI) data were acquired from 24 patients with sd-aMCI, 23 patients with md-aMCI, and 32 healthy controls (HCs). We used the fractional amplitude of low-frequency fluctuation (fALFF) to characterize the intensity of spontaneous brain activity. The analysis of covariance (ANCOVA) and *post hoc* tests was performed to determine the between-group differences in fALFF.

**Results**: We found higher fALFF in left-sided superior-to-middle frontal gyri and middle-to-inferior temporal gyri in sd-aMCI compared to both the md-aMCI and HCs. Conversely, a lower fALFF was found in the left inferior parietal lobe in both the md-aMCI and sd-aMCI patients. The fALFF values in the left middle and inferior temporal gyri were correlated with cognitive performances.

**Conclusion**: The gradual reduction in the left inferior parietal lobe from single to multiple domain aMCI suggest a functional inefficiency underlying cognitive impairment, while increased activity in the frontal and temporal gyri in sd-aMCI rather than md-aMCI might indicate functional compensation. This study indicates differential functional profiles in the sd-aMCI and md-aMCI, which may be helpful for the prediction of the future conversion of aMCI to AD.

## Introduction

Alzheimer’s disease (AD) is a leading cause of dementia worldwide. Once the clinical symptoms of dementia emerge, the brain atrophy has been irreversible, rendering the recognition of AD at the early stage an urgent prerequisite for effective intervention. Amnestic mild cognitive impairment (aMCI) has been considered as a highrisk condition of conversion to AD (Petersen et al., 2001), which is thus a critical stage for early recognition of AD.

The neuropathological basis of aMCI has been investigated extensively. At the stage of aMCI, AD-like patterns of brain change shave been observed, including characteristic patterns of functional and structural atrophies in the temporal and parietal cortices involved in episodic memory (Hu et al., 2014; Farràs-Permanyer et al., 2015). Even though the aMCI diagnosis relies primarily on the presence of memory dysfunction, it is increasingly recognized that aMCI may represent a highly heterogeneous condition and impairments in other cognitive domains were often observed in aMCI patients (Brambati et al., 2009; Lenzi et al., 2011; Li and Zhang, 2015). The aMCI could be classified into single-domain-aMCI (sd-aMCI) and multiple-domain-aMCI (md-aMCI) sub-types (Brambati et al., 2009; Lenzi et al., 2011; Li and Zhang, 2015). The sd-aMCI is characterized by isolated memory impairment, while the md-aMCI is characterized by more widespread cognitive dysfunction involving executive, attention, language, and/or visuospatial abilities (Cid-Fernández et al., 2017). Given that the two sub-types of aMCI have different probabilities of progression to AD (Brambati et al., 2009; Lenzi et al., 2011; Li and Zhang, 2015), it is important to characterize the neural patterns of these subtypes and identify the features that could predict their progression to AD.

However, the relationships between the sd-aMCI and md-aMCI have not been fully clarified. One hypothesis is that the sd-aMCI progresses to md-aMCI, which is an advanced stage toward AD rather than sd-aMCI (Seo et al., 2007; Gu et al., 2019). The sd-aMCI patients were found to have cortical thinning in the left medial temporal lobe relative to healthy controls (HCs), while the md-aMCI patients showed cortical thinning in more widespread regions including the left medial temporal lobe, precuneus, insula, and temporal association cortices (Seo et al., 2007). A combined event-related potential (ERP) and standardized low-resolution brain electromagnetic tomography analysis (sLORETA) study (Gu et al., 2019) found more severe neural deficits during the performance of visuospatial working memory and response inhibition tasks in md-aMCI compared with sd-aMCI patients. In another single-photon emission computed tomography study, reduced metabolism in the fronto-parieto-temporal areas has been observed in both the sd-aMCI and md-aMCI groups, with the md-aMCI group showing additional deficits in the posterior cingulate gyrus (Caffarra et al., 2008). There is also other evidence that the sd-aMCI and md-aMCI reflect two different etiological processes of dementia (Bell-McGinty et al., 2005; Zou et al., 2008; Liu et al., 2017). For instance, the sd-aMCI patients showed more significant volume loss in the left entorhinal cortex and inferior parietal lobe, while the md-aMCI showed larger volume reductions in the right inferior frontal gyrus, right middle temporal gyrus, and bilateral superior temporal gyrus compared to md-aMCI patients (Bell-McGinty et al., 2005). A diffusion tensor imaging (DTI) study reported that the global topological organization of white matter networks was disrupted in patients with md-aMCI but not sd-aMCI (Liu et al., 2017). Our previous study (Zou et al., 2008) of DTI showed decreased FA in the left uncinate fasciculus and left inferior longitudinal fasciculus in sd-aMCI compared to md-aMCI patients. Nonetheless, these studies primarily focused on metabolic and structural differences in patients with sd-aMCI and md-aMCI, how the spontaneous brain activity differs from a regional perspective between both of them remains unknown.

Therefore, we conducted this study to determine the characteristic functional profiles of the subtypes of sd-aMCI and md-aMCI.Neuropsychological and resting-state functional magnetic resonance imaging (fMRI) data were acquired from 24 sd-aMCI patients, 23 md-aMCI patients, and 32HCs. The fractional amplitude of low-frequency fluctuation (fALFF; Zou et al., 2008) was used to measure brain activity intensity. Using this approach, a study has demonstrated that MCI patients had decreased activity in the medial parietal lobe and increased activity in the lateral temporal regions and superior frontal regions (Belleville et al., 2008). Based on these previous functional magnetic resonance imaging (MRI) study in aMCI and the SPECT study comparing the blood flow between sd-aMCI and md-aMCI, we hypothesized that the fALFF changes in both the sd-aMCI and md-aMCI patients would be most pronounced in the posterior temporal regions subserving episodic memory, which has been impaired in MCI and AD patients (Traykov et al., 2007). Givenmore widespread cognitive dysfunction in md-aMCI patients (Albert et al., 2011), the md-aMCI patients may also have functional abnormalities in the prefrontal and parietal cortex. Disrupted brain activity may be associated with cognitive performances.

## Materials and Methods

### Subjects

Twenty-three md-aMCI and 24 sd-aMCI patients were recruited from the Department of Neurology of Xuanwu Hospital, Capital Medical University, between January 2011 and March 2015. Patients were diagnosed according to Petersen’s criteria (Petersen et al., 2001) and National Institute on Aging-Alzheimer’s Association criteria for MCI due to AD (American Psychiatric Association, 1994) according to the criteria: memory complaint; objective memory impairment; near-normal performances on general cognition and preserved daily life activities measured by Activity of Daily Living Scale (ADL); Clinical Dementia Rating (CDR) score of 0.5; failure to meet the criteria of dementia according to the Diagnostic and Statistical Manual of Mental Disorders, fourth edition (DSM-IV; Wang et al., 2011); hippocampal atrophy measured by the Medial Temporal lobe Atrophy scale (MTA scale).

Thirty-two HCs were recruited in the local community. The HCs were cognitively normal and had a CDR of 0, and no history of psychiatric or neuropsychological diseases. All patients had no use of any psychotropic medications for at least 2 weeks prior to the study. The prevalence of vascular factors such as hypertension, hypercholesterolemia, and heart attack did not differ among the three groups of md-aMCI, sd-aMCI, and HCs.

All subjects were Han race and right-handed. Exclusion criteria included neurological illnesses, unstable medical condition, substance dependence or abuse within the last year, a history of electroconvulsive therapy, acutely suicidal or homicidal, current pregnancy or breastfeeding, or any contraindications to MRI scan. The protocol was approved by the institutional review board of Xuanwu Hospital of Capital Medical University. All subjects gave written informed consent in accordance with the Declaration of Helsinki. The demographic and clinical data were provided in Table 1.

**Table 1:** Sample characteristics.

	md-aMCI (*n* = 23)	sd-aMCI (*n* = 24)	HCs (*n* = 32)	*F*	*p*	*Post hoc*
Gender (M/F)	13/10	10/14	14/18			
Age (years)	70.4 ± 8.3	69.8 ± 6.2	67.9 ± 6.4	0.972	0.383	
Education (years)	10.3 ± 3.6	8.3 ± 4.1	11.4 ± 3.6	4.983	0.009	B < A, C
FD	0.23 ± 0.1	0.24 ± 0.1	0.25 ± 0.11	0.397	0.674	
AVLT	4.4 ± 3.6	3.5 ± 2.0	10.9 ± 2.6	99.256	<0.001	A, B < C
MMSE	24.7 ± 3.7	23.9 ± 3.6	28.0 ± 1.9	14.36	<0.001	A, B < C
MoCa	20.4 ± 4.1	19.4 ± 4.6	26.5 ± 2.4	28.995	<0.001	A, B < C
BNT	23.3 ± 2.2	27.9 ± 1.3	29.1 ± 0.7	108.753	<0.001	A < B, C
TMT-A	109.5 ± 19.4	73.1 ± 13.1	54.3 ± 14.2	79.906	<0.001	A > B, C; B > C
TMT-B	222.4 ± 43.3	152.5 ± 38.5	116.1 ± 43.7	40.259	<0.001	A > B, C; B > C

*Data are given as mean ± SD unless otherwise indicated. A, md-aMCI; B, sd-aMCI; C, HC; M, male; F, female; FD, framewise displacement; AVLT, the Auditory Verbal Learning Test; MMSE, Mini-Mental State Examination; MoCA, the Montreal cognitive assessment; BNT, the Boston naming test; TMT, the trail making test. The AVLT, MMSE, MoCA, and BNT were based on numbers correct, and TMT was based on seconds.

### Neuropsychological Tests

All subjects underwent a series of neurological and neuropsychological tests by an experienced neurologist, including mini-mental state examination (MMSE), Montreal cognitive assessment (MoCA), Auditory Verbal Learning Test (AVLT of Chinese version, short delay free recall), Boston naming test (BNT), trail making test (TMT; visual attention and task switching), clock drawing test (CDT; 3-point), and CDR. We used the 3-point CDT to test visuospatial skill, the TMT to test executive function, the BNT to test language skill, and the AVLT (short delay free recall) to test memory ability.

### MRI Data Acquisition

The MRI scans were performed on a 3-Tesla scanner (Siemens Medical Solutions, Erlangen, Germany). The resting-state functional images were obtained using an echo-planar imaging sequence: repetition time (TR)/echo time (TE), 2,000 ms/40 ms; 90° flip angle; matrix, 64 × 64; thickness/gap, 4.0 mm/1.0 mm; 28 slices. The resting-state fMRI scanning lasted for nearly 8 min. For a registration propose, T1-weighted structural images were obtained using a magnetization-prepared rapidly acquired gradient-echo (MPRAGE) sequence: repetition time (TR)/echo time (TE), 1,900 ms/2.2 ms; 9° flip angle (FA); matrix, 224 × 256 × 176; voxel size, 1 × 1 × 1 mm^3^. Before the resting-state scans, subjects were instructed to keep their eyes closed, remain still without head movement, not think of anything in particular, and not fall asleep during the scan. All subjects reported good adherence to these instructions through confirmation immediately after the MRI scans. No subjects showed obvious structural damage based on their MRI images.

### Data Preprocessing

The R-fMRI images were preprocessed with Data Processing Assistant for Resting-State fMRI (DPARSF^1^) based on Statistical Parametric Mapping (SPM12^2^). The first 10 volumes were discarded to allow for magnetization equilibrium. The slice times for the remaining 229 volumes were corrected for different signal acquisition times. The functional volumes were motion-corrected using a six-parameter rigid-body transformation. Subjects with head motion exceeding translation 2 mm or rotation 2° were excluded. The nuisance signals (including Friston 24-parameter model of head-motion parameters, cerebrospinal fluid (CSF) and white matter signals, and linear trend) were regressed out, while the regression of global brain signals was not performed because of a potential induction of negative correlations. Then, derived images were normalized to Montreal Neurological Institute (MNI) space (3 mm^3^ isotropic) using Diffeomorphic Anatomical Registration using Exponentiated Lie algebra (DARTEL) tool. The spatial smoothing was performed using Full Wave at Half Maximum 6 mm.

### The fALFF Computation

We used the fALFF approach to characterize the intensity of intrinsic neural activity. The fALFF analyses were performed using the DPARSF software. Specifically, after the above preprocessing, the fMRI data were temporally band-pass filtered (0.01 < *f* < 0.08 Hz) to reduce the low-frequency drift and high-frequency respiratory and cardiac noise. The time series of each voxel was transformed into the frequency domain, and the power spectrum was obtained. Because the power of a given frequency is proportional to the square of the amplitude of that frequency component, the square root was calculated at each frequency of the power spectrum, and the averaged square root was obtained across 0.01–0.08 Hz at each voxel. This averaged square root was taken as the ALFF, which was assumed to reflect the absolute intensity of brain activity (Zang et al., 2007).

Previous studies have found that although the ALFF reveals significantly higher ALFF in the posterior cingulate cortex, precuneus, and medial prefrontal cortex, other non-specific areas have also been shown to have higher ALFF, such as the cisterns, the ventricles, and/or the vicinities of large blood vessels (Zou et al., 2008), suggesting that the ALFF approach may be sensitive to signal from physiological noise. To overcome these limits, a ratio of the power of each frequency at a low-frequency range to that of the entire frequency range (i.e., fractional ALFF, fALFF) was computed (Di Paola et al., 2009). Specifically, after the removal of linear trends, the time series for each voxel was transformed into the frequency domain without band-pass filtering. The square root was calculated at each frequency of the power spectrum. The sum of the amplitude across 0.01–0.08 Hz was divided by that of the entire frequency range (0–0.25 Hz). The validity of the fALFF approach in suppressing confounding signals from non-specific areas has been confirmed in a sample of healthy subjects (Di Paola et al., 2009).

### Statistical Analysis

#### Between-Group Comparisons in fALFF

One-way analysis of covariance (ANCOVA) was performed to determine the fALFF differences among the md-aMCI, sd-aMCI, and HC groups, controlling for age, gender, educational level, and mean FD values. The results of ANCOVA were corrected for multiple comparisons with a combination of voxel *p* < 0.001 and cluster *p* < 0.05 according to the Gaussian random field (GRF) theory.

Then, for the clusters showing statistical significance during the ANCOVA, we extracted the mean values of the fALFF, and then, performed *post hoc* analyses to determine their differences between two groups within the md-aMCI, sd-aMCI, and HCs by performing 2-sample *t*-tests with statistical significance determined by a Bonferroni-corrected *p* < 0.0083 (0.05/the number of *t*-tests, which is 6).

#### Between-Group Comparisons With GM as Covariates

Structural MRI studies have suggested that the aMCI patients have GM loss in many cerebral regions (Kim et al., 2013). The GM loss may produce partial effects on functional images and thus be a confounding factor for the analysis of functional brain images. We, therefore, re-performed the ANCOVA in the fALFF images among the md-aMCI, sd-aMCI, and HC groups by adding the GM images as covariates.

### Clinical Correlations

The partial correlation analyses were performed between the fALFF mean values of clusters showing statistical significance during ANCOVA and cognitive measures including MMSE, MoCA, AVLT, BNT, TMT and CDT, with gender, age and educational level served as covariates. The statistical significance was determined by an uncorrected *p* < 0.05.

## Results

### Demographic Data

As shown in Table 1, there were significant group differences in years of education, AVLT, MMSE, MoCa, BNT, TMT-A, and TMT-B (*p* < 0.01), while no differences were found in age and gender. The between-group difference in education was mainly driven by the sd-aMCI group. For the AVLT, MMSE, MoCa, BNT, TMT-A, and TMT-B, both the md-aMCI and sd-aMCI groups showed significant decreases compared to HCs (*p* < 0.001). Poorer performances in TMT-A and TMT-B were found in both md-aMCI and sd-aMCI compared to HCs, and in md-aMCI compared to sd-aMCI (*p* < 0.001).

### Between-Group Differences in fALFF

**Figure 1:**
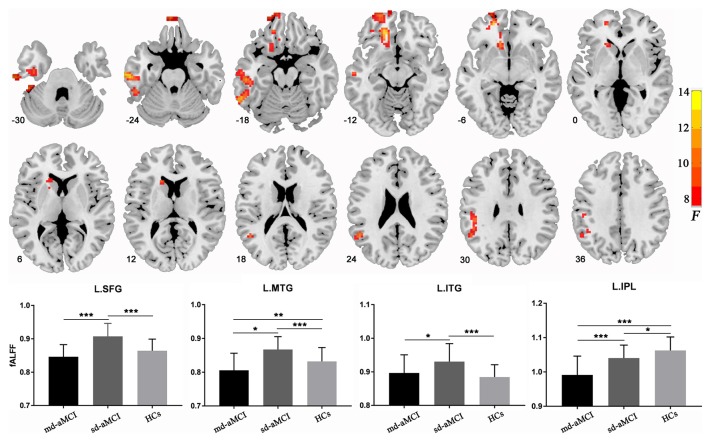
Regions showing between-group differences in fractional amplitude of low-frequency fluctuation (fALFF). The images show a significant group effect on the fALFF in the left frontal gyrus, left middle and inferior temporal gyri, and left inferior parietal lobule. The numbers at the lower-left corner of the image refer to the montreal neurological institute (MNI) coordinates. The bar maps show between-group differences in fALFF in these regions. The data were expressed as mean value ± standard error (SE). *p < 0.05; **p < 0.01; ***p < 0.001.

The ANCOVA among the three groups showed significant group effects in the left-sided superior and middle frontal gyri (peak MNI coordinates: −18, 39, −12), middle (peak MNI coordinates: −63, −18, −24) and inferior (peak MNI coordinates: −39, −15, −33) temporal gyri, and inferior parietal lobe (peak MNI coordinates: −51, −51, 24; Figure 1 and Table 2). For these clusters showing statistical significance during the ANCOVA, the *post hoc* analysis showed higher fALFF values in the left-sided superior and middle frontal gyri, the middle and inferior temporal gyri in the sd-aMCI group than both the md-aMCI and HC groups. Conversely, lower fALFF was found in the left inferior parietal lobe in the md-aMCI than the sd-aMCI group, and in the sd-aMCI than the HC group (Table 2). As shown in Supplementary Figure S1, using the GM volume images as covariates, the fALFF analyses produced results similar to the analyses without GM correction.

**Table 2:** Regions showing between-group differences in fALFF.

Region	Voxels	MNI coordinate (x, y, z)	*F*	*Post hoc*
Superior frontal gyrus/Middle frontal gyrus	287	−18, 39, −12	12.35	B > A, C
Middle temporal gyrus	196	−63, −18, −24	12.799	B > A, C
Inferior temporal gyrus	66	−39, −15, −33	9.198	B > A, C
Inferior parietal lobule	93	−51, −51, 24	9.749	A > B; B > C

*fALFF, fractional amplitude of low-frequency fluctuation; MNI, Montreal Neurological Institute. A, md-aMCI; B, sd-aMCI; C, HC*.

### Clinical Correlations

**Figure 2:**
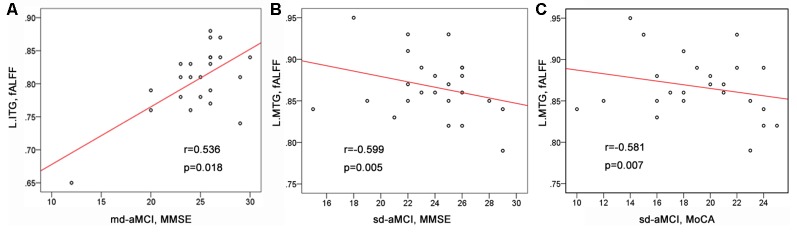
The correlations between the fALFF values and cognitive scores. **(A)** Significant positive correlations between the fALFF of the left inferior temporal gyrus and the mini-mental state examination (MMSE) scores found within the md-Amnestic mild cognitive impairment (aMCI) group. **(B,C)** Significant negative correlations between the fALFF of the left middle temporal gyrus and the MMSE scores, between the fALFF of the left middle temporal gyrus and the MoCA scores found within the single-domain-aMCI (sd-aMCI) group.

As shown in Table 1 and Figure 2, we observed significant positive correlation between the fALFF values of the left inferior temporal gyrus and the MMSE scores within the md-aMCI group. Conversely, a significant negative correlation was observed between the fALFF values of the left middle temporal gyrus and both the MMSE and MoCA scores within the sd-aMCI group.

## Discussion

Using the fALFF in dice, the current study examined the differences in resting-state brain activity in patients with the sd-aMCI and md-aMCI subtypes. The results showed that the sd-aMCI differed from md-aMCI in the fALFF values of the left superior-to-middle frontal gyri, the left middle-to-inferior temporal gyri, and the left inferior parietal lobe. The fALFF in the left temporal gyri were associated with cognitive deficits in aMCI patients. The results suggest that the two subtypes of aMCI may have different functional correlates and the fALFF may be a potential measure to differentiate them.

The first important finding is higher fALFF in the left-sided superior-to-middle frontal gyri and middle-to-inferior temporal gyri in the sd-aMCI than both the md-aMCI and HC groups. The superior and middle frontal gyri are involved in a series of cognitive processes, such as executive function and working memory (Kim et al., 2014; Zamora et al., 2016), while the lateral temporal cortex is more involved in episodic memory (Luo et al., 2018). These cognitive functions are typically impaired in AD and MCI patients (Bell-McGinty et al., 2005; Clément and Belleville, 2010; Teipel et al., 2010; Liang et al., 2011; Scheller et al., 2014; Verfaillie et al., 2016; Melrose et al., 2018). Studies have suggested an involvement of the lateral frontal and temporal cortical regions in aMCI (Bell-McGinty et al., 2005; Clément and Belleville, 2010; Teipel et al., 2010; Liang et al., 2011; Scheller et al., 2014; Verfaillie et al., 2016; Melrose et al., 2018). For instance, a study (Verfaillie et al., 2016) combining the R-fMRI and CSF showed that the regional homogeneity values were associated with the Aβ level of the superior temporal gyrus in patients with sd-aMCI. The thinner temporal cortex has been used to predict the increased risk of future progression to dementia (Teipel et al., 2010). Another study of DTI showed altered fiber integrity in aMCI patients in the callosal genu and anterior midbody connecting the bilateral hemispheres of the prefrontal cortices (Scheller et al., 2014). The GM volume in the superior and middle temporal gyri has been used to differentiate the md-aMCI and sd-aMCI as greater volume loss was observed in patients with md-aMCI compared to sd-aMCI (Bell-McGinty et al., 2005). Despite these structural atrophies, several functional MRI studies (Clément and Belleville, 2010; Liang et al., 2011; Melrose et al., 2018) have shown increased activity in the prefrontal and temporal regions in aMCI patients, which has been considered as a compensatory effect for cognitive impairments. It was proposed that the aMCI patients recruited additional brain regions related to cognitive performances to achieve high demanding cognitive tasks (Clément and Belleville, 2010; Liang et al., 2011; Melrose et al., 2018). Together, higher fALFF in the frontal and temporal gyri in sd-aMCI than both the md-aMCI and HC groups suggest a functional compensation in sd-aMCI patients. It is possible that the sd-aMCI patients may recruit more neural resources from the frontal and temporal regions to compensate for poor cognitive performances and fiber integrity impairment in the bilateral superior and inferior longitudinal fasciculus and left uncinate fasciculus observed in our previous study of DTI (Liu et al., 2017). Further, the behavioral significance of the temporal cortices may be strengthened by significant correlations between the fALFF values of the left temporal cortices and cognitive performances measured by MMSE and MoCA.

In contrast to increased frontal and temporal activities in sd-aMCI patients, we found a gradual reduction in the fALFF values of the left inferior parietal lobe in the md-aMCI and sd-aMCI patients. The inferior parietal lobe is a central node within the posterior DMN, which is highly active at rest but inhibited during goal-directed cognitive tasks (Andrews-Hanna, 2012). The DMN, particularly for the posterior division, plays a key role in highly integrated tasks such as episodic memory (Yi et al., 2015; Sneve et al., 2017). The DMN is among the earliest to show abnormal amyloid deposition and has been the focus of AD and MCI researches (Koch et al., 2015). Most seed-based and independent component analysis of R-fMRI data indicates a loss of connectivity within the DMN in both the aMCI and AD (Dillen et al., 2017; Oishi et al., 2018; Hu et al., 2019). Altered DMN connectivity may be a very early biomarker for AD (Dillen et al., 2017; Oishi et al., 2018; Hu et al., 2019). Reduced GM volume in the inferior parietal lobe has been observed even before the development of aMCI (Oishi et al., 2018). Further, a significant relationship has been found between low GM volume in the right IPL and severity of mental disorientation in aMCI patients (Weise et al., 2018). We, therefore, can speculate that our finding of a gradual reduction in the fALFF of the left inferior parietal lobe from single to multiple-domain cognitive damage may suggest an involvement of the inferior parietal lobe in the neural mechanism of both the single and multiple-domain aMCI.

It is also worthy to note that the fALFF changes in our aMCI patients were predominantlydistributed over the left rather than the right hemisphere. This is consistent with the previous reports of greater amyloid burden on the left hemisphere in aMCI patients (Baron et al., 2001). The VBM studies in AD have demonstrated that the left hemisphere was preferentially affected than the right hemisphere (Karas et al., 2003; Thompson et al., 2003). The right hemisphere may have a “time lag” in developing into structural damage (Oishi et al., 2018). However, the reason underpinning this functional asymmetry needs to be clarified with more targeted research designs.

Despite these important results, several issues need to be further addressed. The small sample may limit the detection of some abnormal brain regions found in previous fMRI studies of aMCI. The structural foundation underlying these brain functional changes remains unclear. Although previous structural MRI studies have suggested the abnormalities of these cortical regions in aMCI (Di Paola et al., 2009; Oishi et al., 2018), a combined analysis of the R-fMRI and structural imaging data (e.g., structural MRI and DTI) will be more helpful for the elucidation of that issue. Our cross-sectional design may limit the assessment of the role of fALFF changes in the subsequent development of dementia. Future prospective studies are warranted to determine how the fALFF of these frontal, temporal and parietal cortices in sd-aMCI and md-aMCI patients change after they convert to AD and how the fALFF differs from the aMCI patients who will be converted and not converted to AD.

In conclusion, we found reduced brain activity in the left inferior parietal lobe but increased activity observed in the left-sided superior-to-middle frontal gyri and the left middle-to-inferior temporal gyri in aMCI patients. These changes may reflect both functional inefficiency and compensation in response to damage at earlier stages of neurodegeneration. These results suggest that the fALFFmaybe sensitive indices for differentiating the sd-aMCI and md-aMCI. Finally, our study demonstrated the neural differences between the sub-types of aMCI from a regional brain perspective and suggests that the md-aMCI might be a more advanced form of the sd-aMCI subtype.

## Data Availability Statement

The datasets generated for this study are available on request to the corresponding author.

## Ethics Statement

The studies involving human participants were reviewed and approved by the institutional review board of Xuanwu Hospital of Capital Medical University. The patients/participants provided their written informed consent to participate in this study.

## Author Contributions

J-HL was responsible for study design. Q-HZ, LW, KW, and X-MZ performed data analysis and article writing.

## Conflict of Interest

The authors declare that the research was conducted in the absence of any commercial or financial relationships that could be construed as a potential conflict of interest.

## Footnotes

^1^http://rfmri.org/DPARSF

^2^http://www.fil.ion.ucl.ac.uk/spm
