# Supplementary material for: Differential Regional Brain Spontaneous Activity in Subgroups of Mild Cognitive Impairment
Source: Front Hum Neurosci. 2020 Jan 30;14:2. doi: 10.3389/fnhum.2020.00002 (PMC7002564; doi:10.3389/fnhum.2020.00002)
Supplement: Supplementary file 1 [file Table_1.DOCX]

**^Supplemental information^**
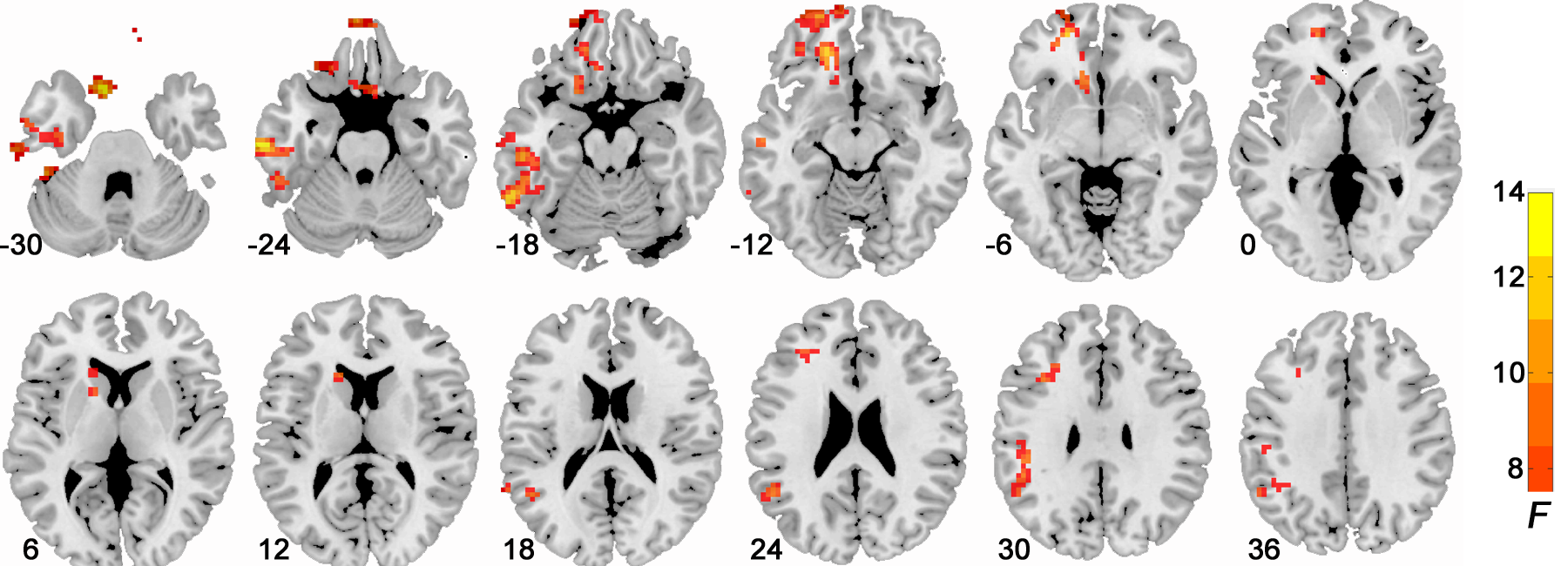


**Figure 1. Between-group differences in fALFF with GM volume as covariates.**

The images show significant group effect on the fALFF among the md-aMCI, sd-aMCI, and HC groups by adding the GM images as covariates.

.
